# Novel Use of Double-Layer Amniotic Membrane Technique in Tube Erosion in a Pediatric Patient

**DOI:** 10.14744/bej.2021.05900

**Published:** 2021-12-17

**Authors:** Altan Atakana Ozcan, Burak Ulas

**Affiliations:** Department of Ophthalmology, Cukurova University Faculty of Medicine, Adana, Turkey

**Keywords:** Ahmed glaucoma valve, amniotic membrane, tube erosion

## Abstract

This case report illustrates the successful use of the double-layer amniotic membrane technique in a child with glaucoma and aniridia. A 3.5-year-old girl with bilateral congenital glaucoma as well as aniridia, lens coloboma, nystagmus, and strabismus had been followed up since birth. Medical treatment did not result in the desired intraocular pressure improvement. An Ahmed glaucoma valve was implanted bilaterally. In a follow-up visit at 2 years of age, there were complaints of redness with watery discharge in the right eye. A biomicroscopic evaluation revealed tube erosion of the conjunctiva. The eroded area was dissected from the surrounding tissue and the area was closed using the new double-layer amnion membrane technique. After the surgery, the intraocular pressure was normal. Subsequent follow-up indicated that the erosion defect was closed and stabilized. The double-layer amniotic membrane technique can be used successfully in cases of conjunctival tube erosion. To the authors' knowledge, this is the first published pediatric case report of using the novel technique of amniotic membrane transplantation for tube erosion.

## Introduction

Glaucoma may present with aniridia and lens coloboma in pediatric ages. Glaucoma drainage implants have become a popular method of controlling intraocular pressure in pediatric glaucoma (1, 2). Ahmed glaucoma valve implantation technique is effective to controlling the intraocular pressure (2). However, glaucoma drainage surgery in pediatric patients is associated with complications (1-3). The common complications are tube-cornea touch, hypotony, choroidal detachment, capsule fibrosis, ocular motility disturbance, and tube erosion (1-3). However, exposure of the tube through overlying eroded conjunctiva is the most common shunt-specific complication which can lead inflammation, hypotony, phthisis, and endophthalmitis (1-3). A lots of patch graft material (sclera, dura, amnion, pericardium, fascia lata, and cornea) have been used for tube erosion (3, 4). Any of these methods is superior to another for providing durability in the follow-ups (5).

We present a congenital aniridia case of tube erosion and explain our approach new technique of double-layer amniotic membrane in pediatric age.

## Case Report

**Figure 1.**
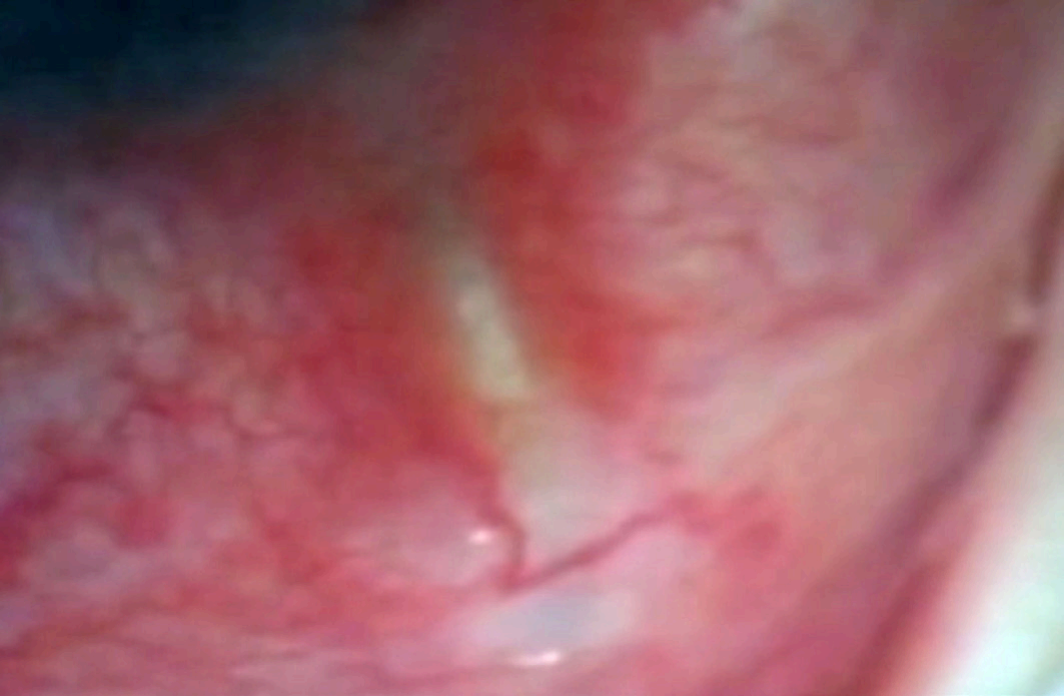
Erosion of the conjunctiva over the Ahmed glaucoma valve.

This study was conducted in accordance with the principles of the Declaration of Helsinki and an informed consent form was completed by the patient’s family. A 3 and 5-year-old girl with bilateral congenital glaucoma who was also suffering from aniridia, lens coloboma, nystagmus, and strabismus was followed up since birth. Bilateral intraocular pressures were 30 mmHg. The cup-to-disc ratio was 0.9 in bilateral fundus examination. Intraocular pressures did not reach the desired level with full medical treatment. For the patient in question, Ahmed glaucoma valve implantation was evaluated and the surgery was performed bilaterally. In follow-ups, intraocular pressures were regulated in both eyes (14 mmHg in the right and 15 mmHg in the left eye) and the corneal thickness was 700 microns at post-operative 12^th^ month; in the right eye, conjunctival erosion was seen 4 mm away from the limbus over the tube at the post-operative 24^th^ month (Fig. 1).

**Figure 2.**
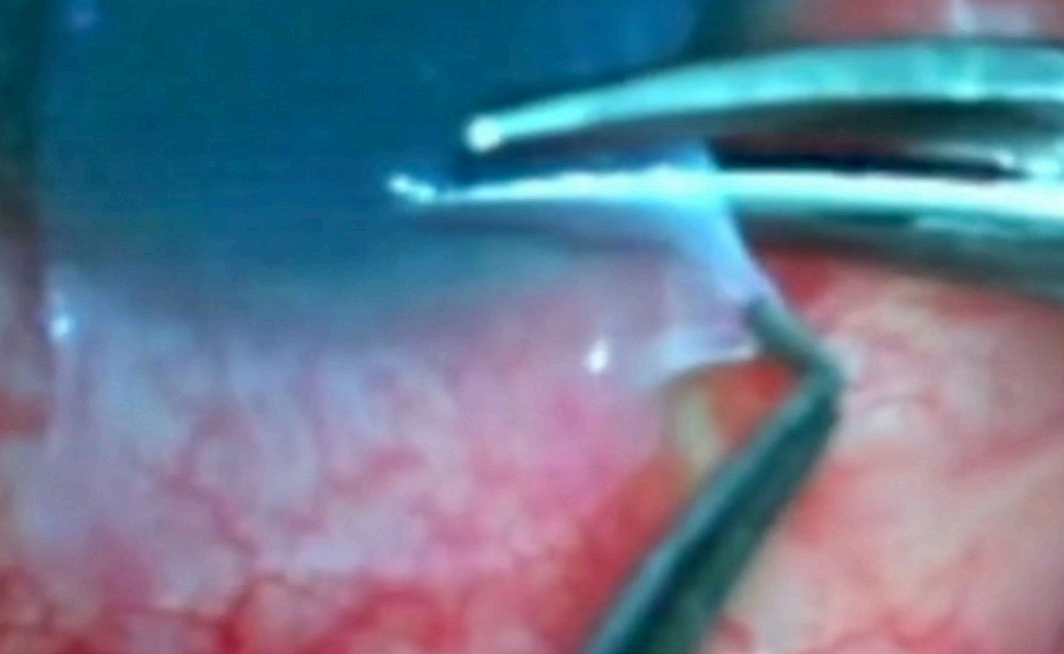
It was seen that the first layer of amniotic membrane is applied over the tube with the epithelial side.

**Figure 3.**
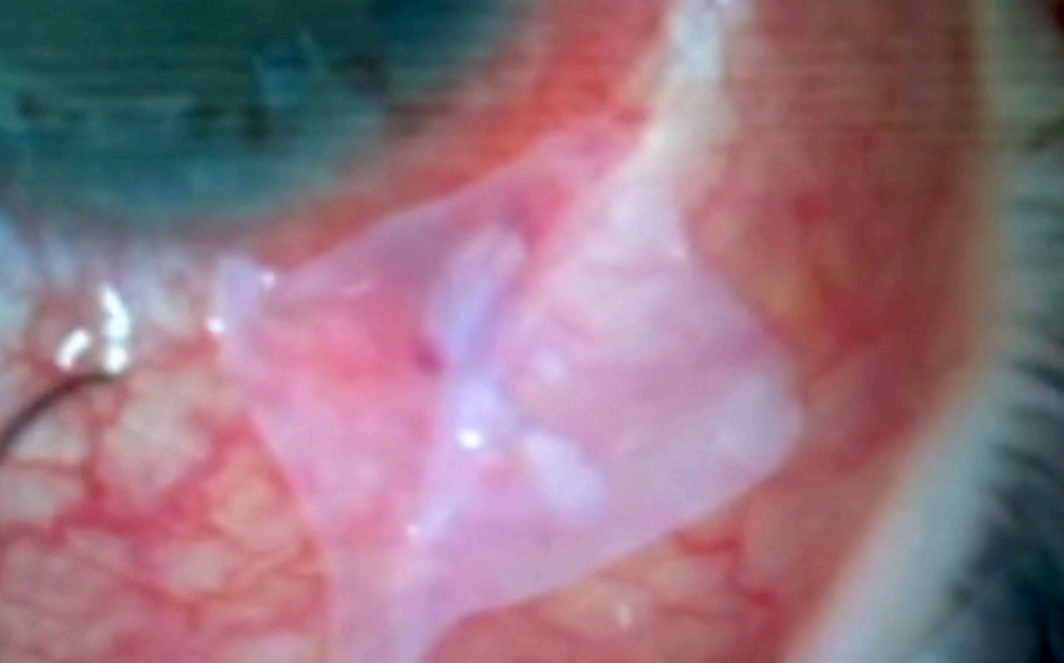
A second layer of amniotic membrane is then applied over the epithelial defect.

The area affected by conjunctival erosion was dissected from the surrounding tissues and the conjunctival opening was closed with a double-layer amnion membrane. After the first step in closing the anterior composite conjunctiva-Tenon flap and bringing it back to its original position, the amniotic membrane’s first layer was applied over the area of conjunctival tube erosion with the epithelial side up (Fig. 2). The amniotic membrane’s second layer was placed epithelial side down to conjunctival epithelial defect which functioned as a patch to protect the underlying membrane (Fig. 3). One month later following the amniotic membrane transplantation, the conjunctival tube erosion defect was closed and intraocular pressure was within the normal range. In the 1-year follow-up, no erosion defect recurrence was tracked and intraocular pressure was stable.

## Discussion

Aqueous shunt surgery is effective to control intraocular pressure in glaucoma patients (6). The use of shunt devices for glaucoma management has increased dramatically in the recent years (3, 6). Complications related to shunt surgery include post-operative hypotony, capsule fibrosis, tube erosion, and infection (6, 7). Amniotic membrane has been used in glaucoma for the repair of tube erosions (4, 6-8). This new technique is as described in material method; double amniotic membrane layer covers the conjunctival tube erosion defect (4). Ainsworth et al. (4). described the management of tube erosion with double layer amniotic membrane technique in three adult patients. The underlying epithelial side up membrane provides support for epithelial migration from the conjunctiva and closure of the erosion site. The second amniotic membrane layer is applied onto the epithelial defect. This technique has been depicted before and has seen successful long-term results (4).

In pediatric ages, glaucoma may seen with aniridia and lens coloboma and glaucoma drainage implants are the good at controlling intraocular pressure in pediatric glaucoma. Glaucoma drainage implant complications seem to be more common and serious in children because children may have lifelong problems about these complications. Exposure of the tube through overlying eroded conjunctiva is the most common and undesirable shunt-specific complication (9). This technique provides an additional approach to management of pediatric glaucoma surgery complications (4). In our pediatric case, we particularly considered using this technique which provided good results in tube erosion. To the best of our knowledge, this case is the first in the literature in terms of repairing the conjunctival defect with a double-layer amniotic membrane in a pediatric case. The limitation of our study is that it is by now the only pediatric case. Thus far, a lot of patch graft material have been used for tube erosion, still our new technique of double-layer amniotic membrane provided good result in pediatric age. We believe it would be beneficial to continue studies with more pediatric cases.

Even though many techniques and materials are successfully used for tube erosion management, further comparative studies might be needed to determine the best repair method. If tube erosion occurs, this new double-layer amniotic membrane technique could provide good results regarding the treatment of tube erosion at pediatric ages.

### Disclosures

**Informed consent:** Written informed consent was obtained from the patient for the publication of the case report and the accompanying images.

**Peer-review:** Externally peer-reviewed.

**Conflict of Interest:** None declared.

**Authorship Contributions:** Involved in design and conduct of the study (AAO); preparation and review of the study (AAO, BU); data collection (AAO, BU); literature review (BU); writer (AAO, BU); critical review (AAO).
